# Efficient Radiation by Electrically Small Antennas made of Coupled Split-ring Resonators

**DOI:** 10.1038/srep33501

**Published:** 2016-09-15

**Authors:** Liang Peng, Peiwei Chen, Aiting Wu, Gaofeng Wang

**Affiliations:** 1 Key Laboratory for RF Circuits and Systems (Hangzhou Dianzi University), Ministry of Education, Hangzhou, 310018, China; 2 Department of Electronic Engineering and Information Science, Hangzhou Dianzi University, Hangzhou, 310018, China

## Abstract

In this paper, coupled split-ring resonators (SRRs) are used to construct the electrically small antennas. We show that through strong magnetic coupling, the coupled SRRs composite can oscillate at a wavelength much larger than its total size. Due to its magnetic dipole feature, the coupled SRRs composite allows the electromagnetic (EM) power to radiate and hence forms the electrically small antenna (ESA). Because of the high-Q resonance, the ESA could be easily matched to the driving circuit in the microwave region, through mutual induction approach. We also demonstrate that the radiation efficiency of such ESAs can be drastically improved if the current distribution on individual SRRs is similar, which is achievable by carefully designing the ESAs. From our simulations and experimental measurements, the ESAs’ radiation efficiency can reach up to 41%, with relative footprint of 0.05*λ*_0_ × 0.05*λ*_0_. Our approach would be an effective way to realize ESAs with high efficiency, which can be implemented on chip through the standard planar lithography.

Nowadays, the electrically small antennas(ESAs) are attracting people’s eyes, due to their reduced size and the capability to be integrated on chip. For the ESAs, efficient radiation occurs when the operating frequency matches the antennas’ resonance. As the ESAs are generally small, the bandwidth is limited, which had already been explained by Wheeler and Chu^1^,^2^. Meanwhile, the ESA’s radiation resistance is commonly much smaller than the Ohmic loss on the radiating elements, thus the radiation efficiency is suppressed. Traditional on-chip ESAs often apply spiral metallic structures^3^,^4^,^5^,^6^, which usually makes the bandwidth and the loss issues worse.

To improve the performance of the ESAs, efforts are made to find new approaches in the design and fabrication. From antenna fundamentals, to increase the available relative bandwidth, one should increase the ESA’s capacitance and suppress its inductance. Moreover, to reduce the Ohmic resistance, an ESA would adopt short-bold metallic strucutres, rather than those long-thin wire structures, like helix or spirals. Magnetic metamaterials, which employs simple subwavelength split-ring resonators (SRRs), are suggested to be a good candidate to fulfill these two requirements^7^,^8^,^9^,^10^. In ref. 7, ESA consists of a monopole and a circular split-ring resonator structure, with footprint 0.095*λ*_0_ × 0.1*λ*_0_, has a gain up to 2.38 *dBi* with 43.6% efficiency. In ref. 8, the electrically small antenna made of SRR shrinks to a footprint of 0.06*λ*_0_ × 0.06*λ*_0_, whose gain reaches −1.302 *dBi* under radiation efficiency 21.4%. The complementary SRR structure shows its ability to further reduce the footprint when applied in designing the ESAs^9^,^10^. In refs 11, 12, 13, 2D and 3D ESAs with either electric or magnetic resonance show low port reflection, without applying any matching network. Meanwhile, by using two SRRs with different size, the ESA would be dual banded with a compact structure^14^.

In this context, we propose the ESA design through multiple coupled SRRs. Our ESA consists of a rectangular feeding loop and several radiating SRRs. To realize strong magnetic coupling, the SRRs are identical, and are aligned and stacked together. Due to the strong magnetic coupling, the magnetic dipole resonance of the whole ESA can be effectively shifted to a frequency, which is much lower than that of the individual SRR. By carefully controlling the SRRs, the current distribution on each SRR would be similar, which helps to reduce the Ohmic loss and to enhance the radiation, i.e. to promote the radiation efficiency drastically. In the simulations and experiments, six ESAs are designed to work around 500 MHz. Possessing footprint of 3 *cm* × 3 *cm*, equivalently 0.05*λ*_0_ × 0.05*λ*_0_, these ESAs can radiate EM power up to 41%, a drastical improvement compared to any other existing ESAs of similar relative footprint as we know. Through our approach, the ESAs can have a compact size, and are easily fabricated through the standard planar techniques.

## Results

**Figure 1:**
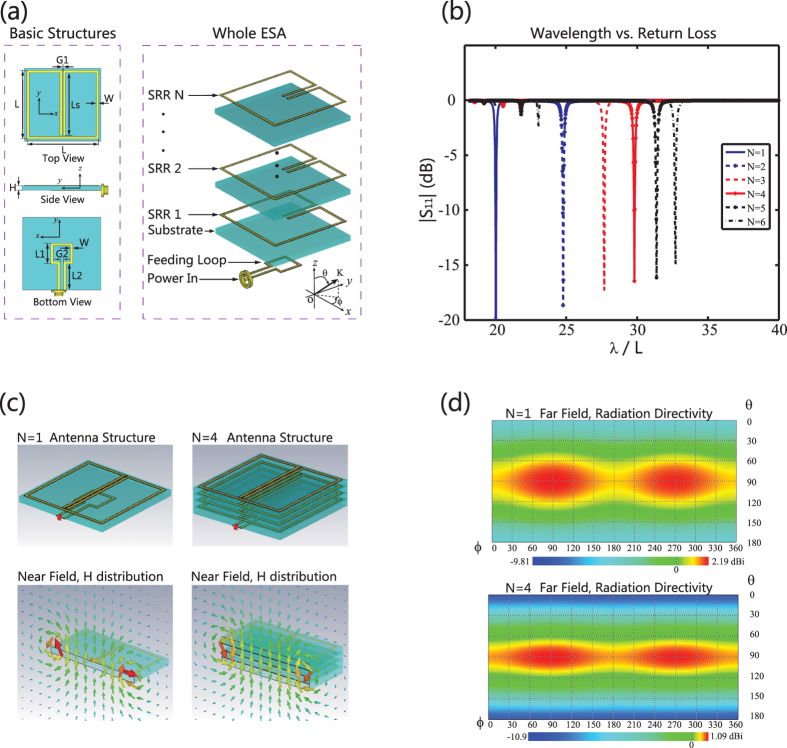
(**a**) The geometry and construction of the proposed ESAs. In the design and simulation, *L* = 30 mm, *L*_1_ = 9 mm, *L*_2_ = 11.5 mm, *G*_1_ = 0.37 mm, *G*_2_ = 1.5 mm, *W* = 1 mm, *H* = 2 mm. (**b**) The return loss of the SRR-ESAs with different *N*. (**c**) The magnetic field distribution in the antenna region, it is seen that the magnetic resonances resembles a standard dipole. (**d**) Far field pattern of the *N* = 1 and *N* = 4 ESAs. It is seen that the radiation is omnidirectional in the horizontal plane. The unbalanced design in SRRs makes the radiation pattern slightly distorted. In (**b**–**d**), *L*_*s*_ = 27.8 mm.

The basic structure of our ESA is made of an SRR, together with a small feeding loop, as referred to Fig. 1(a). The proposed antenna is fabricated on a 2 mm thick PTFE substrate (F4T, or Rogers RT5880), whose dielectric constant is 2.2 and a loss tangent of 0.0009. The SRR with magnetic resonance is placed on the top of the substrate, while the feeding loop is set in the backside.

The two branches, being set at the center of the SRR (with length *L*_*s*_ and gap *G*_1_), work as the capacitor and bring capacitance into the SRR loop. Basically, to lower the SRR’s resonant frequency, we shall increase the length *L*_*s*_ and/or reduce *G*_1_. However, to make the SRR to be electrically small is somehow difficult due to two main reasons. First, in the standard planar technology, i.e. printed circuit board (PCB) or the optical lithography, *G*_1_ has its limitation. Second, *L*_*s*_ can not exceed the SRR’s largest size (*L*).

In order to effectively lower the ESA’s resonant frequency, here we introduce strong magnetic coupling into the ESA by adding additional SRRs, as shown in Fig. 1(a). In Fig. 1(a), *N* identical SRRs are coaxially aligned and stacked together. The space in-between two adjacent SRRs is filled by the 2 mm thick F4T substrate. We emphasize that this multi-layered structure is realizable in the multi-layered PCBs and the 3D integrated circuits^15^,^16^.

In the simulation, the ESA made of a single SRR works at wavelength of 20 *L*. Since the SRRs oscillate at frequencies much lower than that of the feeding loop, the coupling between the feeding loop and the SRRs will not drastically influence the ESA’s overall resonance. For the two-SRR ESA, the working wavelength is around 24.6 *L*. Please refer to the solid and the dash-doted curves in Fig. 1(b). The lowest resonant frequency (LRF) of the two-SRR ESA may be tuned by changing the coupling coefficient (*κ*), i.e. change the interval between the two SRRs. We emphasize that the resonant frequency of the ESA may be further shifted down by placing more identical SRRs into the antenna system, as the direct outcome of the coupled mode theory, see the Method section. Here, rather than addressing too much theoretical formulation, we do the simulations for the ESAs with multi-SRR configuration, see Fig. 1(b). As we see that the ESAs’ LRF decreases as the number of the SRRs increases, i.e. with six SRRs, the ESA’s LRF is found around 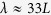.

In Fig. 1(b), we see that the ESAs with different SRR number are perfectly matched to the driving circuit, without applying any additional matching network. This is mainly because of the high quality (high-Q) resonance of the ESAs, due to which the induced current on the separated SRRs will be enhanced, hence the input power is efficiently radiated into the outer space, or consumed by the Ohmic resistance of the metals. As a result, the ESAs are resistive. Due to the mutual coupling, the equivalent resistance of the ESAs on the feeding loop is alternated^17^,^18^. In our cases, the size of the feeding loop makes impedance matching eventually happen. In case the impedance is not well matched, the size of the feeding loop is tuned.

As an intrinsic property, the bandwidth of an ESA with superior performance is generally limited. In our study, the relative bandwidth (|*S*_11_| < −10 *dB*) of all the ESAs are less than 0.3%. Again, due to the small sized SRRs, all the ESAs behave as short magnetic dipoles with similar radiation profiles. The near field(H-field) distribution and the radiation pattern, of the ESAs with *N* = 1 and *N* = 4, are shown in Fig. 1(c,d).

### Current flow and Radiation Efficiency

**Figure 2:**
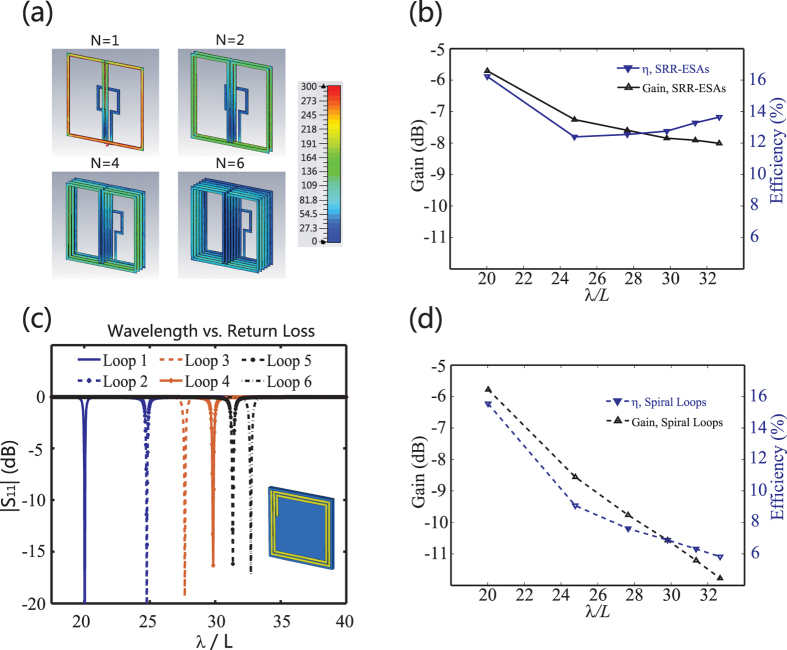
(**a**) Current distribution in *N* = 1, 2, 4, 6 cases. It is found that the current intensity is degraded as *N* grows, as the effect of strong magnetic coupling. For all the four cases, the current distribution on the SRRs of a single ESA is approximately similar. (**b**) The gain and efficiency of the proposed SRR-ESAs. (**c**) The return loss of the spiral ESAs for comparison. The schematic of the spiral is shown as the inset of this figure. The footprint of all the spirals is identical to the one-SRR ESA, as well as the thickness and dielectric constant of the substrate. For all the spirals, the strip is made of cooper with 1 mm width, the distance between adjacent strips is 0.5 mm. In realizing different working frequencies, the turns of the spirals is tuned. (**d**) The gain and efficiency of the spiral ESAs.

The current distributions for the ESAs with *L*_*s*_ = 27.8 mm are shown in Fig. 2(a). We see that the current intensity on each SRR deduces as *N* increases, which is ensured by the strong magnetic coupling. Again due to the strong magnetic coupling, the current distribution on each SRRs of a single ESA is approximately similar. As a result, the Ohmic loss on the metals is deducted. Please go to the Method section for details. The overall antenna gain and efficiency are exhibited in Fig. 2(b). We could see that although the electric size of the ESAs shrinks (Fig. 1(b)), the efficiency of the six ESAs does not drastically drop, unlike the conventional planar ESAs.

Here, to better illustrate the advancement of our SRR ESAs, we simulate the radiation of six planar spiral antennas as comparisons. In the design, the spiral ESA’s footprint, the substrate’s thickness and dielectric constant are identical to those of the one-SRR ESA. The basic topology of the spiral ESAs is illustrated as the inset of Fig. 2(c). The number of turns for each spiral ESA is tuned, so that these six spiral ESAs possess working frequencies the same as the aforementioned SRR-ESAs. The |*S*_11_| curves of the spiral ESAs are exhibited in Fig. 2(c), and the corresponding gain and efficiency are displayed in Fig. 2(d). From Fig. 2(b,d), the performance promotion of our SRR-ESAs is obvious. By the way, in case the substrate’s thickness of each spiral ESA is tuned to be the same as the corresponding SRR-ESA, the improvement of efficiency is less than 1%, due to the fact that the resonance of the spirals is localized and little influenced by the substrate.

**Figure 3:**
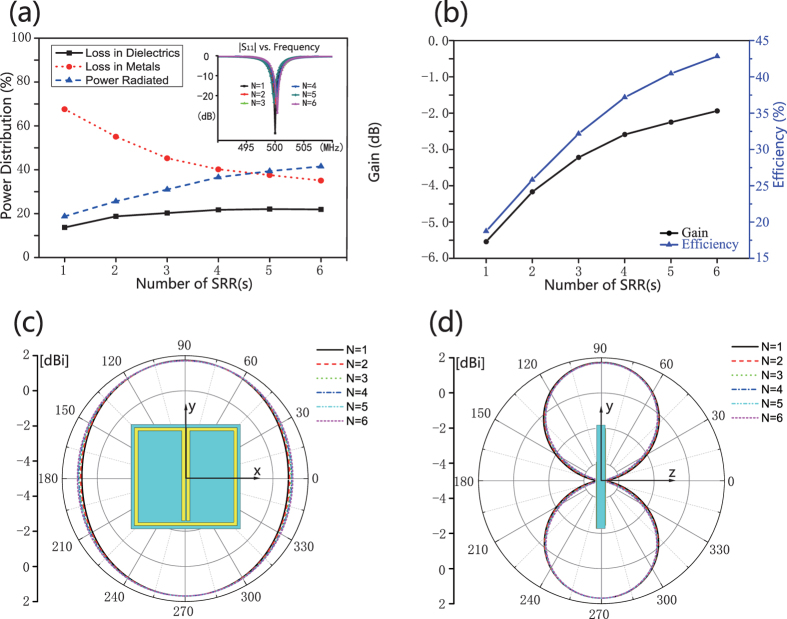
The simulated results for the six ESAs with the same working wavelength. (**a**) The ratio of the power dissipation, i.e. *P*_*res*_, *P*_*d*_ and *P*_*rad*_. (**b**) The simulated antenna gain and radiation efficiency of the six ESAs. (**c**,**d**), the radiation pattern in the azimuthal and the elevation planes, respectively.

From Fig. 2(b), it is also observed that *η* slowly grows up as *N* increases in *N* ≥ 2 cases, i.e. the ESAs’ electric size shrinks. We would image that the radiation efficiency may be drastically improved if the ESAs resonant at the same wavelength. To show this, we tune the antennas’ operation frequency the same, which is accomplished by shrinking *L*_*s*_ for each ESAs. Here, the operation wavelength for all the six ESAs is 20 *L*, i.e. same as the one-SRR ESA. In *N* = 2, 3, 4, 5, 6 cases, *L*_*s*_ are 16.87 mm, 12.98 mm, 10.67 mm, 9.21 mm and 8.18 mm, respectively. Figure 3(a) shows the ratio of the power consumed in metal (*P*_*res*_), substrate (*P*_*d*_) and radiation (*P*_*rad*_). The deduction of *P*_*res*_ is observed when *N* ≥ 1, as we predict. Thus both the gain and the efficiency can be promoted, see Fig. 3(b). The radiation directivity of all the ESAs are also shown in Fig. 3(c,d). It is seen that the minor changing in antennas’ dimensions does not alternate the radiation pattern too much, due to the magnetic dipole resonant profile of all the ESAs.

### Experimental measurement

**Figure 4:**
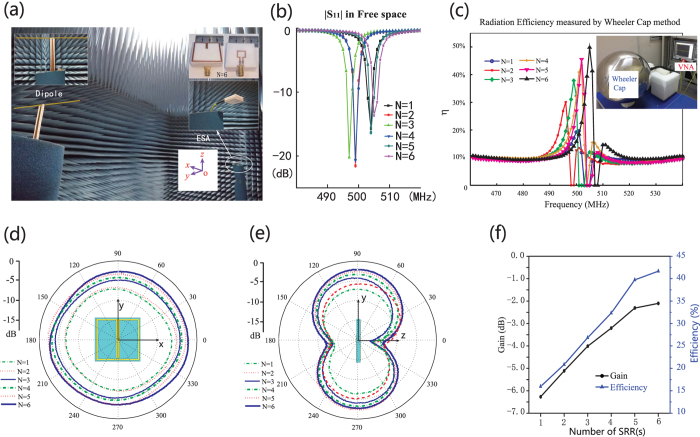
(**a**) The experimental measurement of the ESAs. The standard half wavelength dipole and the *N* = 6 ESA are exhibited as the two insets. (**b**) The measured return loss of the six ESAs. (**c**) The efficiency measured by Wheeler cap method. The experimental setup is exhibited as the inset of this figure. (**d**) The radiation pattern in the *x*-*y* (horizontal) plane. (**e**) The measured radiation pattern in the *y*-*z* (vertical) plane. (**f**) The measured gain and the retrieved efficiency, of the proposed ESAs. In calculating the antenna efficiency, the forward 
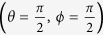
 directivity of all the six ESAs is chosen as 1.7 dBi, a number obtained from the simulated results shown in Fig. 3(c,d).

In the experiments, the six ESAs refer to Fig. 3 are fabricated. The measurement of the ESAs’ radiation is conducted in the microwave anechoic chamber, see Fig. 4(a). The copper SRRs are fabricated on the 2 mm thick F4T substrate, e.g. the photograph of the six-SRR ESA can be found in the inset of Fig. 4(a).

First, the port reflectance by the six ESAs is recorded, as shown in the Fig. 4(b). We see that all the six ESAs work at around 500 MHz, an equivalent wavelength of 20 *L* as designed. Although the working frequencies of the fabricated ESAs deviate the designed frequency slightly, their radiation is not influenced drastically.

The radiation efficiency of the six ESAs is measured through the modified Wheeler cap method^19^,^20^. The setup of the Wheeler cap is shown as the inset of Fig. 4(c). The efficiency extracted from the measured S-parameters is exhibited in Fig. 4(c). We see that as *N* increases, the radiation efficiency gradually grows. However, we could notice that the efficiency curves are seriously influenced, i.e. a sudden drop always occurs near the resonance. This is induced by the secondary resonance produced by the SRRs together with the Wheeler cap, whose resonance is very close to the working frequency of the ESAs^21^. Moreover, the efficiency for each ESA is overestimated by the Wheeler cap method, compared to the simulated results in Fig. 3(b).

In measuring the radiation profile the ESAs, a standard half-wavelength dipole antenna is used to receive the radiated power, and helps to calibrate the ESAs’ gain, see the inset of Fig. 4(a). With the aid of the dipole, the radiation pattern of the ESAs is recorded, as shown in Fig. 4(d,e). Noticing the directivity curved in Fig. 3(c,d), the antenna efficiency is reasonably retrieved, see Fig. 4(f). The improvement in efficiency from below 17% to above 41% is observed, as expected by our simulation.

## Discussion

Theoretically, in the lowest resonance mode (the fundamental mode) of the SRR-ESA, the ESA radiates like a magnetic dipole, and the magnetic field inside the SRR region is almost uniform, see Fig. 1(c). Hence, the current on each SRR behaves in the similar manner. Ideally, all the current loops contribute equally to the total induced current on the feeding loop. As we see that the ESAs are well matched, the current on the feeding loop should be identical for each ESA. Thus the current intensity on each SRR of a single ESA would be identical. Hence we could simply conclude that the current density on each SRR decreases as the number of SRRs, *N*, increases, provided the coupling between the single SRR and the feeding loop is identical, otherwise the port current grows and the ESAs are mismatched. In fact, this effect is well supported by our simulated results in Fig. 2(a), in which the current intensity on each SRR is degraded when *N* becomes large.

To secure effective radiation, the shrinking of current intensity on each SRR when *N* increases, is crucial. The reason is two folded. First, the Ohmic loss would be effectively deducted, in case the current intensity is suppressed. Please go to Method section for more information. Second, due to the reduced current intensity, the electric field strength in the substrate region decreases. Thus although the volume occupied by the ESA is enlarged with large *N*, 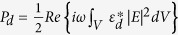 will not drastically grow up. Particularly, *P*_*d*_ may be much smaller than the Ohmic loss, provided the substrate is low loss or lossless, hence would be safely neglected for the common ESAs.

## Methods

### Coupled Mode Analysis

For simplicity, we assume that the ESA made of *N* (*N* > 1) SRRs is divided into two resonators, i.e. the first one with (*N* − 1) SRRs (resonant at *ω*_1_), and the second one with only one SRR (resonant at *ω*_2_). The electromagnetic dynamics of the ESA made of two radiating elements is described by the coupled mode equations^17^,^22^,^23^,^24^, which read


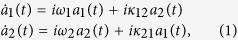


where the indices denote the different resonators. The variables *a*_*m*_(*t*) (*m* = 1, 2) are defined so that the energy contained in the *m*-th resonator is |*a*_*m*_(*t*)|^2^, respectively. In our case, *ω*_1_ < *ω*_2_, and *κ*_12_ = *κ*_21_ = *κ* being the coupling coefficient in-between the two resonators.

The eigen-frequency of the two-SRR antenna could be found by solving Eq. (1). Here we rewrite Eq. (1) in a matrix form, which reads


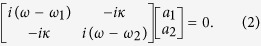


It is obvious that the system possess two eigen resonances, with 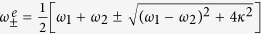. For the first eigen-mode, 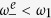, indicating the low shift of the ESA’s fundamental mode. Particularly, when the two resonators are placed very close to each other, *κ* would be a number comparable to *ω*_1_, then the working frequency of the ESA would be drastically reduced, equivalently the electric size of the ESA would be further shrinked. For the second eigen-mode, 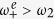, it does not help in reducing the antenna size, so we do not placed too much discussion on it.

### Radiation Efficiency Estimation

For the antennas, the total power provided by the source is expressed as *P*_*total*_ = *P*_*res*_ + *P*_*d*_ + *P*_*rad*_, with *P*_*res*_ being the Ohmic loss on the metals, *P*_*d*_ being the power dissipation in the surrounding dielectrics (substrate), and *P*_*rad*_ being the power radiated. The radiation efficiency (RE) is evaluated through 
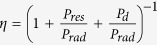
.

From classical electromagnetics, we find 
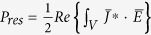
, 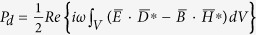 and 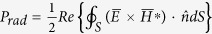, where *S* is the closed surface of an arbitrary volume *V* containing the antenna^25^. The Ohmic loss is the summation of the resistive loss on individual SRRs, i.e. 
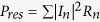
, with *I*_*n*_ and *R*_*n*_ being the *n*-th SRR’s current and resistance. The *P*_*rad*_ can be evaluated through the radiated field. According to ref. 26, the radiated field is expressed as


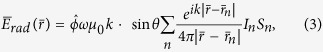


where 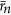 is the position and *S*_*n*_ is the area, of the *n*-th SRR, respectively. We assume that the intervals between different SRRs are so small that the phase involved by 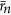 can be safely neglected, then 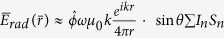. Consequently, *P*_*rad*_ can be estimated by integrating 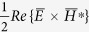 over a sphere of radius *r* with *r*− > ∞. Hence we have 
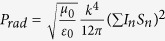
. Synthesizing *P*_*res*_, *P*_*d*_ and *P*_*rad*_, we derive the radiation efficiency as


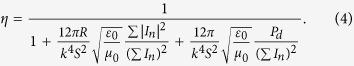


Here, we use *R*_*n*_ = *R* and *S*_*n*_ = *S*, for all the SRRs are identical. The dominating term in Eq. (4) is the one involving 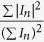. As a special case, if the current on each SRR is identical, i.e. *I*_*n*_ = *I*, one have 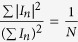, which is meaningful for high *η*.

To excite identical current distribution on each separate radiating element, the SRRs are aligned and coaxially stacked together, like those periodically arranged metamaterial unit cells^27^, which is crucial in maximizing the coupling coefficient and stimulating the effective radiation. However, in stacking the SRRs, one should notice that if the interval between adjacent SRRs (*H*) is small or comparable to the width of the SRR strip (*W*), the localized modes in-between the adjacent SRRs are excited, which in turn would significantly affect the current flow on separated SRRs. Empirically, we have 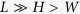 in the design.

### Experimental Setup for Radiation Measurement

As the feeding cable may distort the radiation pattern due to the leakage current, the experimental setup is carefully arranged. In measuring the radiation pattern, the ESAs are horizontally placed, i.e. *x*-*y* plane lies horizontally, but the feeding cable is confined in the *x*-*z* plane (polarized vertically). To effectively detect the E-field radiated by the ESA alone, which is in *ϕ* direction, the reference dipole is horizontally polarized. In Fig. 4(a), we highlight the reference dipole in yellow color to make it clear.

With the aforementioned experimental setup, we would conclude that the radiation of the leakage current may influence the side radiation level, i.e. (*θ* = *π*/2, −*π*/2 < *ϕ* < *π*/2) or (*θ* = *π*/2, *π*/2 < *ϕ* < 3*π*/2), but leaves the forward level (*θ* = *π*/2, *ϕ* = *π*/2) almost undisturbed, since the horizontally polarized dipole is no aware of the vertically polarized radiation from the leakage current. Suppose power consumed on the ESA is *P*_*a*_, power radiated by the ESA is *P*_*ar*_, power consumed on the feeding cable is *P*_*c*_, and power radiated by the cable is *P*_*cr*_. The radiation efficiency by the whole system is 
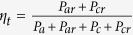
. The radiation efficiency of the ESA alone is 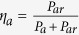. The radiation efficiency of the leaky cable is 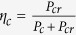. Generally, the leaky cable is longer than the side size of the ESA, so it commonly radiates more efficiently than the ESA, i.e. *η*_*a*_ < *η*_*c*_. The efficiency measured through Wheeler cap method is *η*_*t*_, which is larger than *η*_*a*_ and makes the efficiency be overestimated. In Fig. 4(f), we use the forward (*θ* = *π*/2, *ϕ* = *π*/2) power level, with a pattern factor of 1.7 dB for reference, to estimate the radiation efficiency, which is not the efficiency of the whole system, but may be exactly interpreted as 
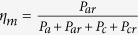
. Clearly, this operation would less estimate the radiation efficiency of the practical antenna system. However, in our study, we focus our eyes on the improvement of the radiation efficiency from the ESAs, the increment of the measured efficiency is something reflecting the justification of our design, since the leakage power is commonly somehow much less than that consumed on the ESAs.

## Additional Information

**How to cite this article**: Peng, L. *et al*. Efficient Radiation by Electrically Small Antennas made of Coupled Split-ring Resonators. *Sci. Rep.* **6**, 33501; doi: 10.1038/srep33501 (2016).
